# Hypoxic Inducible Factor Stabilization in Pericytes beyond Erythropoietin Production: The Good and the Bad

**DOI:** 10.3390/antiox13050537

**Published:** 2024-04-27

**Authors:** Dario Troise, Barbara Infante, Silvia Mercuri, Claudia Piccoli, Bengt Lindholm, Giovanni Stallone

**Affiliations:** 1 Nephrology, Dialysis and Transplantation Unit, Advanced Research Center on Kidney Aging (A.R.K.A.), Department of Medical and Surgical Sciences, University of Foggia, 71122 Foggia, Italy; 2 Renal Medicine and Baxter Novum, Department of Clinical Science, Intervention and Technology, Karolinska Institutet, 141 52 Stockholm, Sweden; 3 Department of Clinical and Experimental Medicine, University of Foggia, 71122 Foggia, Italy

**Keywords:** pericytes, ischemia–reperfusion injury, hypoxia, hypoxia inducible factors (HIFs), endothelial cells (ECs), fibrosis

## Abstract

The paracrine signaling pathways for the crosstalk between pericytes and endothelial cells are essential for the coordination of cell responses to challenges such as hypoxia in both healthy individuals and pathological conditions. Ischemia–reperfusion injury (IRI), one of the causes of cellular dysfunction and death, is associated with increased expression of genes involved in cellular adaptation to a hypoxic environment. Hypoxic inducible factors (HIFs) have a central role in the response to processes initiated by IRI not only linked to erythropoietin production but also because of their participation in inflammation, angiogenesis, metabolic adaptation, and fibrosis. While pericytes have an essential physiological function in erythropoietin production, a lesser-known role of HIF stabilization during IRI is that pericytes’ HIF expression could influence vascular remodeling, cell loss and organ fibrosis. Better knowledge of mechanisms that control functions and consequences of HIF stabilization in pericytes beyond erythropoietin production is advisable for the development of therapeutic strategies to influence disease progression and improve treatments. Thus, in this review, we discuss the dual roles—for good or bad—of HIF stabilization during IRI, focusing on pericytes, and consequences in particular for the kidneys.

## 1. Introduction

Pericytes (PCs) play a pivotal role in the control of physiological and pathophysiological processes in different organs and vascular beds [1]. PCs exhibit molecular differences and exert different functions depending on their anatomical location, development origins, and modes of vessel recruitment [2]. Coordinated endothelial cell (EC)–PC interactions are required to facilitate vascular remodeling during hemodynamic changes. These interactions are mediated by coordinated responses between soluble mediators, such as transforming growth factor-β (TGF-β), platelet-derived growth factor-β (PDGF-β), vascular endothelial growth factor (VEGF), angiopoietin 1 and 2 (Ang1/Ang2), and their receptors [3].

The paradoxical increase in cellular dysfunction and death that occurs when blood flow to previously ischemic tissues is restored is known as ischemia–reperfusion injury (IRI). IRI may occur in different organs, such as the kidney, brain, heart, lung, gut, and skeletal muscle, starting a chain of events possibly resulting in multi-organ failure. Local and systemic inflammatory responses, oxidative stress and production of reactive oxygen species (ROS) lead to cellular apoptosis, a recognizable characteristic alteration that occurs cin damaged tissues after IRI. Moreover, hypoxic insults increase the expression of several genes, including hypoxia-inducible-factor-1 (HIF-1), VEGF, glucose transporter type 1 (GLUT1), and many others that have a significant impact on how cells react to hypoxia [4].

Although PCs are considered the major kidney erythropoietin (EPO) producing cells through HIF-dependent gene transcription, and HIF stabilizers are emerging as promising treatments for anemia in chronic kidney disease (CKD) patients [5], PCs are also considered the major precursor cells of myofibroblasts in CKD and a source of fibrosis in the kidney [6].

Currently, the impact of HIF stabilization on PCs during IRI and its effects beyond EPO production is still poorly understood. HIF stabilization is intended as a stable expression of HIF target genes responsible for maintaining biological homeostasis in response to decreased oxygen availability [7]. This review emphasizes the dual consequences of HIF activity during IRI focusing on PCs and their role in vascular remodeling and fibrosis in CKD.

## 2. Pericytes (PCs)—Endothelial Cells (ECs) Crosstalk

PCs are interstitial fibroblast-like cells embedded within the basement membrane of micro-vessels in close contact with endothelial cells (ECs). Their long processes surround the endothelial wall and are typically connected with ECs via adhesion plaques and via peg–socket junctions, providing support to the vasculature. Peg–socket junctions are characterized by PC cytoplasmic fingers (pegs) that are inserted into endothelial invaginations (pockets), whereas adhesion plaques are junctions formed by bundles of microfilament containing fibronectin that anchor these two cells [8]. Through these specific connections, a single PC often connects with more than one EC, which allows PCs to integrate and coordinate nearby cell responses. Depending on the vascular bed, PC’s coverage of ECs ranges from approximately 10% to 50%, and the main vascular sites where they were found are blood capillaries, arterioles, and venules. The central nervous system (CNS) has the highest PC coverage, particularly in the retina where the relative frequency of PCs to ECs is 1:1, whereas the skeletal muscle has a lower rate (1:100). Consequently, the retina appears to be the most vulnerable site for partial pericyte loss. Moreover, PC morphology varies among different organs. For example, in the kidney, they are rounded, compact, and only focally attached to the basement membrane, whereas in the CNS, they are flattened or elongated with multiple cytoplasmic processes, in contact with a large abluminal vessel area [9].

**Figure 1:**
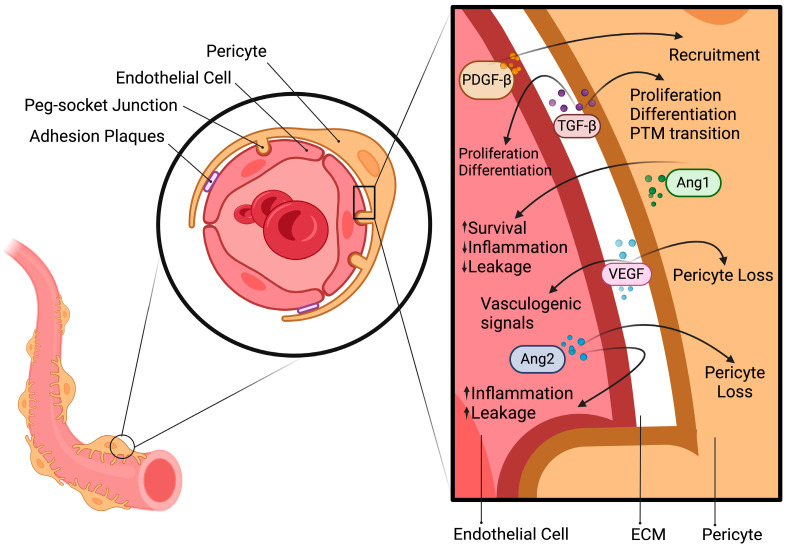
Pericyte and endothelial cells coordinated responses. In both healthy individuals and during pathological conditions, PC-EC crosstalk is critical for developing coordinated responses between cells. Paracrine signaling is regulated by the production of regulatory factors involved in vascular development, inflammation, and cell differentiation. PDGF-β: platelet-derived growth factor-β; TGF-β: transforming growth factor-β; PTM: pericyte-to-myofibroblast; Ang1/Ang2: angiopoietin 1 and 2; VEGF: vascular endothelial growth factor. Created with BioRender.com.

The paracrine signaling pathway between PCs and ECs is essential for the development of blood vessels in healthy individuals and under pathological conditions. Angiogenesis is initiated when blood vessels experience hypoxia and inflammation, causing the production of relevant angiogenic factors, such as VEGF and Ang2. In response to Ang2, PCs detach from the vessel wall due to protein hydrolysis mediated by matrix metalloproteinase allowing the release of inactive VEGF from the extracellular matrix (ECM). Subsequently, one EC is chosen to differentiate into a leading cell, known as a tip cell, placed at the tips of vascular sprouts that coordinate multiple processes during angiogenesis and lead to the migration of new ECs. This process is regulated by various signaling pathways and molecules; HIF-1α is one of them. During neovascularization, the recruitment of PCs is essential for preserving normal blood vessel morphology and promoting basement membrane deposition after the connection between ECs and PCs is established [10]. Angiopoietins are regulatory factors involved in the control of vascular development. Ang1 is produced by PCs and participates in maintaining EC survival by inhibiting inflammation and vascular leakage. On the other hand, Ang2 is produced by activated ECs and is involved in inflammation and vessel leakage, acting as an antagonist of Ang1 [11,12]. TGF-β is synthesized by PCs and ECs as a latent complex with its prodomain and is maintained in the ECM in its inactive state. Therefore, upon direct interaction between PCs and ECs, TGF-β is activated. TGF-β is synthesized with large amino-terminal pro-domains that confer latency [13] and cocultures of ECs and PCs were showen to induce the activation of TGF-β [14]. During the activation process, one of the most significant intracellular modifications involves the cleavage of the C-terminal pro-region from the N-terminal portion of the protein due to pH variations, heat, chaotropic agents and other physiological substances, such as serine protease, plasmin, neuraminidase, and cathepsis [15]. Activated TGF-β transduce a signal through activin receptor-like kinases 1 and 5 (Alk1–Alk5) and SMAD 2/3/5. Alk1 is preferentially expressed on ECs and mediates proliferation to promote angiogenesis, whereas Alk5 is expressed on both ECs and PCs, mediating proliferation, and differentiation [16,17,18]. Moreover, another important signaling pathway in EC-PC communication is the platelet-derived growth factor (PDGF)/PDGF Receptor-β (PDGFR-β) pathway. It affects PC development, proliferation, and recruitment during angiogenesis [19,20]. Compensatory overexpression of VEGF-A, the proliferation of ECs and abnormal junction development are induced by the loss of the PDGF-/PDGFR-β signaling pathway [21]. VEGF expression by PCs and other perivascular cells is a crucial element in the initiation of angiogenesis and can be considered as a survival factor between ECs and PCs. Transmembrane tyrosine kinase receptors for VEGF are expressed mainly by ECs; they are known as VEGFR1, VEGFR2 and VEGFR3 and are responsible for vasculogenic signaling transduction and EC migration [22,23,24]. VEGFR1 is also expressed by PCs and has been shown to mediate PC loss, as stated by Cao et al. [25] (Figure 1).

## 3. Ischemia–Reperfusion Injury and HIF

**Figure 2:**
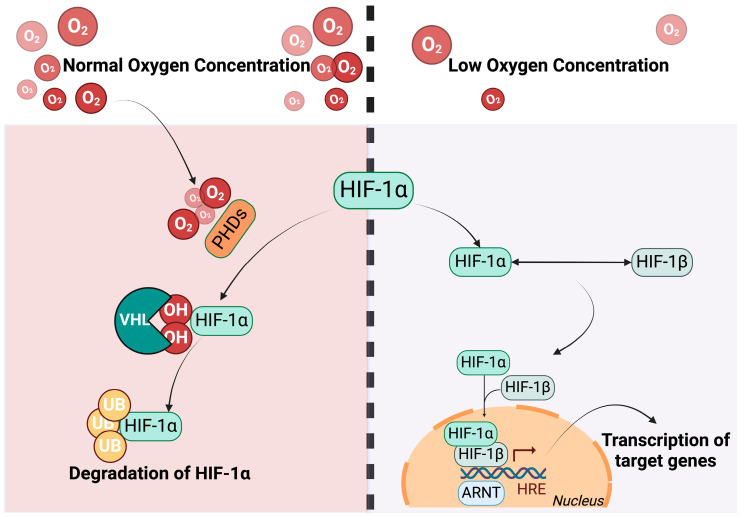
Regulation of HIF-1α depending on O_2_ concentration. When the oxygen levels are normal HIF-1α undergoes prolyl hydroxylation on its proline residues by PHDs. This process facilitates its degradation, orchestrated by the VHL through the ubiquitin proteasome pathway. In conditions of low oxygen concentration, the VHL tumor suppressor protein becomes inactive, enabling the formation of a heterodimer between HIF-1α and HIF-1β in the nucleus. This heterodimer assembles into a transcriptional activation complex that binds to the HRE and acts as a transcription factor. Created with BioRender.com.

Hypoxia leads to the activation of hypoxia-inducible transcription factors (HIFs), which are heterodimers formed by an α subunit (O_2_-sensitive) and a β subunit (O_2_-insensitive) and include three distinct members known as HIF-1, HIF-2, and HIF-3. Moreover, three different genes encode for three identified HIF-α subunits (HIF-1α, HIF-2α, HIF-3α) that dimerize with the same β subunit (HIF-1β) in humans [26]. Among these, HIF-1α is the most well known and mediates the most adaptive changes in response to hypoxic environments in different organs while HIF-2α is considered to have a role in angiogenesis, lipid metabolism regulation, cell migration, and tumor invasion. HIF-3α showed the capacity to both inhibit and activate the HIF pathway, depending on the HIF-3α isoform. HIF-1α has a short half-life (5 min) because the von Hippel–Lindau tumor suppressor protein (VHL) is responsible for its degradation after recognition by prolyl hydroxylase proteins (PHDs), which serves as an oxygen sensitivity system leading to HIF-1α hydroxylation and its subsequent ubiquitination in normoxic conditions [27]. When the O_2_ concentration reaches dangerously low levels, PHDs are inactivated and the heterodimers can translocate to the nucleus where they form a transcriptional complex with co-activators and bind to hypoxia response elements (HREs), inducing the activation of target gene transcription [28]. The effects of HIFs are difficult to evaluate. Normoxic conditions led to the rapid degradation of HIF, increasing the difficulty in precise assessments of its protein levels in tissue. Moreover, the same HIF isoform can regulate distinct target genes in various cell types [29,30] (Figure 2).

Under conditions of low oxygen concentration, most eukaryotic cells are able to shift their metabolic processes from predominantly mitochondrial respiration to glycolysis to support adequate production of ATP. Increased glycolysis is useful for maintaining bioenergetic homeostasis but may also have a significant influence on hypoxic endothelial and immune cell biological functions and tumor development [31], as well as on defective pericyte-endothelial cell interactions [32]. Evidence shows that HIF-1-mediated activation of glycolysis is essential for tissue metabolic adaptation to hypoxia because it increases the conversion of glucose to pyruvate and then lactate to maintain ATP levels and prevent ROS production [33]. Therefore, glycolysis helps to restore the high-energy phosphate level after reperfusion [34]. Matsushima et al. have investigated the role of NADPH oxidases 2 and 4, the heart’s major Nox isoforms, and showed that they mediate myocardial IRI through the production of ROS and oxidative stress, but they also play a crucial part in HIF-1α regulation in response to ischemia–reperfusion insults through the inactivation of PHD and subsequent upregulation of HIF-1α with a potential protective role against IRI [35].

Mitochondrial impairment is a major feature of ischemia insults. Increased intracellular Ca^2+^ is found in low O_2_ level conditions, arising even more during reperfusion, leading to a calcium overload, and opening of mitochondrial permeability pores. In the heart, both Ca^2+^ levels and ROS production contribute to cardiomyocyte apoptosis or necrosis [36]. Na^+^/H^+^ exchanger-1 (NHE1) plays a well-established role in regulating intracellular pH in cardiomyocytes by producing a significant transmembrane exchange of Na^+^ and H^+^ ions, allowing the removal of excess acidity from the cytoplasm [37]. During ischemia, NHE1 may become activated in response to intracellular acidosis resulting from anaerobic metabolism and during reperfusion, when the transmembrane pH gradient is at its peak. The activation of NHE1 has been associated with increased mitochondrial oxidation, Ca^2+^ overload and ROS levels in animal models [38]. HIF-1 was shown to increase the mRNA and protein expression of NHE1 in pulmonary arterial smooth muscle cells, mediating vascular remodeling during hypoxic insults [39].

HIF-1α could transcriptionally control the levels of frataxin, which acts as a cardioprotective factor against IRI. Frataxin is a mitochondrial protein that regulates mitochondrial Fe-S cluster formation during the oxidative phosphorylation process and plays a role in iron storage during conditions of iron overload, providing an essential antioxidant function by reducing the production of ROS during iron excess. Evidence shows that HIF-1α expression increases frataxin levels, thus reducing ROS generation and mitochondrial iron overload and protecting the mitochondrial membrane from damage [40]. Moreover, compared with the control myocardium, higher levels of HIF-1α have been found in the myocardium IRI model and they were related to the expression of microRNAs (miRNAs). The relationship between mitochondria and the cell nucleus is essential for the stability of these organelles, and miRNAs play a pivotal role in this mitochondria–nucleus dialogue. Low levels of miR-138 are related to myocardial IRI. Overexpression of miR-138 demonstrated a cardioprotective role in decreasing the infarct size by inhibiting mitochondria-mediated apoptosis by targeting HIF-1α [41]. Song et al. stated that miR-126 expression may be induced by HIF-1α in endothelial cells after myocardial infarction, which promotes angiogenesis in peri-infarct areas through regulation of the activity of the phosphatidylinositol 3-kinase/protein kinase B pathway (PI3K/AKT) [42]. Moreover, Li H.S. et al. showed that hypoxic-mediated apoptosis is attenuated by HIF-1α which exerts a role in the expression of genes that control mitochondrial fission and fusion, such as GTPase Drp1 levels and optic atrophy factor 1 (Opa1) levels [43]. Mitochondrial fission is essential for cardiac homeostasis; it separates the mitochondrion into two daughter mitochondria and allows the removal of damaged organelles from the healthy network for degradation by selective autophagy of the mitochondria [44]. Mitophagy may occur through ubiquitin-dependent or -independent mechanisms. The independent mechanism is mediated by different receptors, among which BNIP3 (BCL2/Adenovirus E1B 19 KDa Protein-Interacting Protein 3) has been reported to be upregulated via HIF-1α, leading to the clearance of damaged mitochondria, and promoting myocardial remodeling to provide cardiac protection after IRI [45]. The production of mitochondrial antioxidants in cells is also promoted by HIF-1α through the nuclear factor erythroid 2-related factor 3 (Nrf2) and by increasing the synthesis of antioxidants such as glutathione and superoxide dismutase 2 [46].

### 3.1. HIF-1α and Cerebral IRI

Cerebral ischemic injury leads to neuronal cell death after sudden rupture of cerebral vessels, cerebral artery embolism, or thrombosis, resulting in decreased blood supply in specific areas of the brain. Endogenous substances, including amino acids and neurotransmitters, have a neuroprotective role against cerebral ischemia by controlling HIF-1α. For example, after IRI in rats, arginine and glycine can reduce the inflammatory response mediated by HIF-1α and protect neuronal cells from death [47,48]. Increased expression of VEGF and HIF-1α have been linked to increased levels of the α7 nicotinic acetylcholine receptor, which promotes the formation of cerebral arteries and reduces cerebral ischemic damage [49]. Moreover, Jin et al. showed that cavin-1, a cytoplasmic protein involved in the signal transduction pathways and participating in bioenergetic processes, was increased in neuronal cells after IRI and that its expression was regulated by the signal transducer and activator of transcription 3 STAT3/HIF-1α axis, whose inhibition could help to reduce the infarct volume and the neurological deficits in IRI models [50]. However, some studies proposed that HIF-1α could contribute to cerebral IRI by inducing pro-inflammatory cytokine production. Yang et al. showed that nitric oxide may promote cerebral ischemia/reperfusion injury through upregulating HIF-1α-associated inflammation processes and apoptosis in rats [51].

### 3.2. HIF-1α and Renal IRI

The kidneys are considered one of the organs most vulnerable to hypoxic injury because of the intricate functional interplay between oxygen consumption, renal blood flow, and glomerular filtration rate (GFR). IRI is considered a well-known risk factor involved in the pathogenesis of acute kidney injury (AKI) [52]. In renal IRI, mitophagy mediated by the HIF-1α-BNIP3 axis has demonstrated a protective role through the inhibition of oxidative stress and apoptosis in tubular cells [53], as well as miR-668 HIF-related expression, which inhibits pathological mitochondrial fragmentation [54]. Moreover, the induction of miR-21 and VEGF by HIF is essential in the neovascularization process after ischemic kidney damage and may be related to the inhibition of thrombospondin 1, a multifunctional protein recognized as an angiogenesis inhibitor [55]. The inflammatory response during renal IRI is regulated by numerous transcription factors. Nuclear factor kB (NF-kB) plays a pivotal role and is closely linked to HIF-1α at transcriptional levels. Li et al. found that NF-kB is required for enhancing HIF-1α transcription in renal tubular epithelial cells [56].

### 3.3. HIF-1α and Hepatic IRI

In the liver, the interleukin-1 receptor antagonist (IL-1ra) expression is considered a pivotal factor in the regulation of hepatic IRI. Evidence has shown that higher levels of IL-1β and tumor necrosis factor-α (TNF-α) are released in the hepatic tissue after ischemic and reperfusion insults, leading to the secretion and activation of several proinflammatory cytokines and pathways. IL-1ra, a naturally endogenous IL-1 inhibitor that binds to the IL-1 receptor without eliciting a signal, can significantly reduce hepatocyte damage during IRI-induced proinflammatory cytokine production [57]. By interfering with iron homeostasis, the Fenton reaction between ferrous iron and ROS causes iron-dependent necrosis, called ferroptosis, leading to excessive lipid peroxidation and cell death in a HIF-1α-dependent way during IRI. In hepatic tissues, ferroptosis can be inhibited by the activation of the µ-opioid receptor (MOR), which exerts a hepatoprotective role by reducing liver dysfunction and inflammation via p53 expression [58]. However, in animal models, HIF-1α overexpression is linked to the upregulation of adenosine receptors, among which the A2B adenosine receptor (A2BAR) is widely distributed throughout the heart, liver, lungs, kidneys, and blood vessels and is involved in tissue adaptation to hypoxia and inflammation. Evidence has shown that the expression of A2BAR is induced by HIF-1α in endothelial cells, dendritic cells, and cancer cells after hypoxic damage. A2BAR activation in hepatic IRI attenuates NF-kB signaling and consequent liver cell inflammation, protecting the liver from damage [59].

## 4. Ischemia–Reperfusion Injury and Pericytes

Renal IRI causes disruption of the integrity of ECs, leading to loss of peritubular capillaries and hypoxia, which is considered a primary initiator of fibrotic alterations in the kidney. In this context, PCs can detach from the endothelium and differentiate into myofibroblasts after migrating to the interstitium, contributing to kidney fibrosis. Khairoun et al. showed that IRI in rats causes a loss of ECs, PC proliferation, and the development of fibrosis linked to an imbalance between Ang2 and Angiopoietin1 (Ang1) [60]. Their findings have also been validated in the field of transplantation, in which reperfusion of kidney allografts has led to increased levels of Ang2 [61]. Human myofibroblasts in patients with CKD show increased expression of tumor endothelial marker 1, known as CD248 or endosialin. This marker is a PC type I transmembrane glycoprotein, and increased expression was linked to poor renal survival [62]. Using a renal IRI murine model, CD248 was found to be upregulated in renal myofibroblasts, as observed by Pai et al. Moreover, CD248 knockout rats exhibited decreased renal fibrosis and macrophage recruitment due to a reduction in myofibroblast collagen production [63]. PCs play a role in regulating kidney medullary blood flow; they are associated with the descending vasa recta and cortical and medullary peritubular capillaries and show expression of α-smooth muscle actin (α-SMA) which mediate the contraction of capillary PCs and controls the distribution of blood flow within the renal medulla [64]. After ischemia and reperfusion, α-SMA was found to be strongly expressed by PCs surrounding the descending vasa recta, resulting in capillary constriction, presumably via actomyosin-based contractility. Moreover, another key modulator of actin polymerization and PC contraction is the Rho kinase pathway, which increases phosphorylation of the myosin light chain by inhibiting myosin phosphatase, thus increasing PC contraction. Rho kinase blocking after ischemic insult and from the start of reperfusion reduced acute kidney injury through inhibition of PC contraction and increased medullary blood flow [65]. Metabolically, mitochondria are largely responsible for ATP generation in renal PCs, despite evidence showing that glycolysis represents the principal metabolic pathway responsible for the production of ATP in proliferative placental PCs [66], suggesting that different PC subpopulations rely on different metabolic pathways probably due to different oxygen supplies. According to Chen et al., the pericyte-to-myofibroblast (PTM) transition mediated by TGF-β1 is characterized by increased glycolysis and elevated phosphorylation levels of serin threonine kinase mTOR. The inhibition of the PI3K-Akt-mTOR pathway resulted in decreased glycolysis, suggesting that this pathway plays a role in the regulation of PTM [67]. These results are consistent with studies in AKI animal models obtained by IRI. Researchers have observed increased expression of the pyruvate kinase M2 subtype (PKM2) in renal-damaged PCs, suggesting that glycolysis is a pivotal metabolic pathway for PTM [68]. Therefore, targeting PC metabolic reprogramming during the AKI to CKD transition can prevent PTM, as stated by Xu et al. They showed that the enhancement of fatty acid oxidation or the inhibition of the glycolytic pathway during renal IRI in an animal model can influence the fate of PC transdifferentiation and prevent the progression of the damage [69].

**Figure 3:**
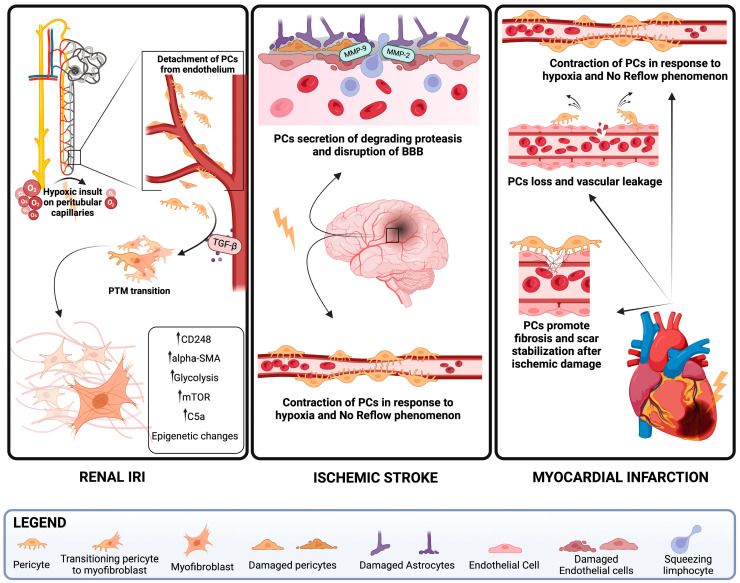
Ischemic insults leading to loss of pericytes and fibrotic damage. Schematic illustration showing how IRI leads to damage of pericytes in the kidney, brain, and heart. In the kidney, after ischemic insult, PCs can detach from the endothelium of peritubular capillaries and undergo phenotypical, metabolic, and epigenetic changes under the influence of cytokines and other pro-inflammatory factors. These changes are defined by the pericytes-to-myofibroblast transition. In the brain, ischemic stroke is associated with disruption of BBB and the “no-reflow” phenomenon. PCs contribute to the damage in both phenomena with secretion of ECM-degrading proteases and sustained reversible constriction of their associated capillaries. In myocardial infarction, PCs are dysfunctional, leading to higher vascular leakage, no-reflow, and contributing to scar stabilization. Created with BioRender.com.

In renal IRI, the complement system plays a crucial role in mediating tissue damage and enhancing innate and adaptive immune responses. Specifically, C5a exhibited a profibrotic action by modulating the TGF-β pathway through ERK activation, leading to increased early interstitial extracellular matrix deposition and the acquisition of PTM phenotype, suggesting that PCs are one of the targets of complement activation during IRI [70]. Microarray analyses were conducted by Chou et al. who showed that epigenetic modifications during IRI are present in PCs and contribute to the shift into a profibrotic and proliferative phenotype, leading to the progression of CKD and increased fibrogenesis [71]. Decreased vascular relaxation following reperfusion can lead to a “no-reflow phenomenon”, which is characterized by higher resistance of microvascular blood flow after an occluded blood artery reopens during infarct-related insult and is linked to poor outcomes [72]. One of the leading causes of disability worldwide is ischemic stroke, defined as an acute-onset condition caused by the occlusion of a cerebral artery. Nowadays, reperfusion of the ischemic tissue obtained from the recanalization of the occluded vessel is considered the treatment of choice. However, “no-reflow” of the cerebral microvasculature could result in further tissue damage. PCs have been shown to play a role in this phenomenon because they are implicated in sustained reversible constriction of their associated capillaries, which has detrimental effects on cerebral blood flow during and within the first 24 h post-stroke, as demonstrated by Shrouder et al. [73]. Therefore, during the acute phase of brain ischemia, oxidative stress leads to the accumulation of neurotoxic substances and subsequent impairment of BBB integrity and PCs start to secrete degrading proteases, such as matrix metalloproteinase-2 and -9 (MMP-2, MMP-9), which induce their migration and cellular damage resulting in BBB disruption. However, PCs may also contribute to the maintenance of BBB through regulation of the development of cerebral microcirculation after their recruitment to the vessel walls in the embryonic brain, as stated by Winkler et al. [74]. Regarding microvascular blood flow, PC contraction after reperfusion is induced by oxidative-nitrative stress and may be the cause of the failure of thrombolytic therapy in stroke. Moreover, immune-inflammatory responses may be driven by PCs through the production of pro-inflammatory factors such as chemokines and cytokines, which can amplify the inflammatory process in the brain after ischemic stroke and disrupt BBB functioning [75]. Myocardial IRI and subsequent microvascular dysfunction lead to a decreased rate of normal myocardial reperfusion [76]. Cardiac PCs can quickly contract in response to ischemia, and after the reperfusion, they may not relax, which might lead to no-reflow phenomena and PC apoptotic rigor [77]. Furthermore, PCs from ischemic hearts showed a senescent phenotype along with signs of oxidative stress, decreased angiogenic potential, and impaired ribosome biogenesis [78,79]. Vascular hyperpermeability results from PC dysfunction and loss and is associated with the development of fibrotic lesions and impairs communication between surrounding cardiomyocytes and stromal cells. In addition, this can cause left ventricular systolic and diastolic dysfunction. Moreover, the expression of TGF-β was high in PCs post-myocardial infarct. One to seven days after the ischemic damage, PCs migrated to the injury site and expressed fibrotic-related genes to promote fibrosis and stabilize scar tissue [80]. However, after the production of pro-inflammatory mediators and recruitment of neutrophils, macrophages, and other immune cells in the early phase after ischemia, PCs start to produce immune regulatory molecules that help to reduce the acute inflammatory responses, such as leukemia inhibitor factor, cyclooxygenase 2, haemooxygenase 1, and IL-6 [81]. These studies suggest a pivotal role for PCs in IRI (Figure 3 and Table 1).

## 5. HIF Stabilization in Pericytes

**Figure 4:**
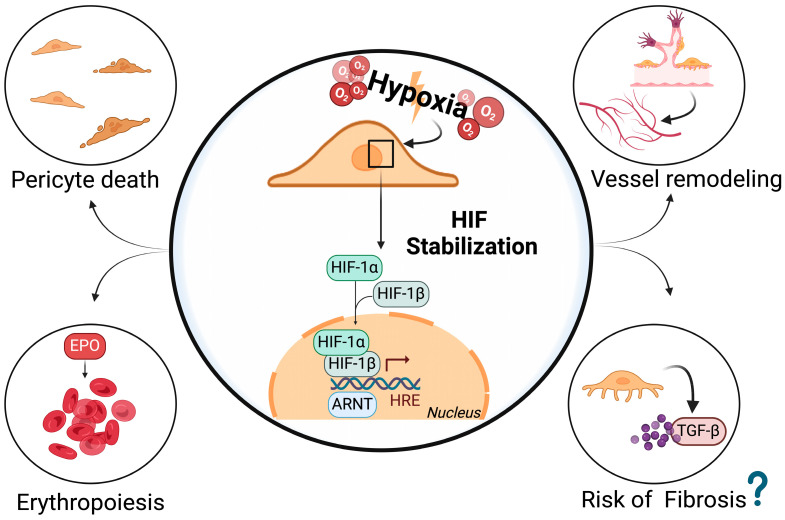
HIF stabilization in pericytes. This figure illustrates a summary of features linked to HIF-1α stabilization in pericytes on vascular remodeling, erythropoiesis, and pericyte loss. The effects of HIF stabilization on fibrosis are still poorly understood. Created with BioRender.com.

During hypoxia in the normal kidney, PCs produce EPO through the binding of the HIF2α/HIFβ complex to the 5′HRE located at 9248 bp upstream of the EPO transcriptional start site, thereby initiating EPO transcription [85]. Anemia is largely caused by impairment of production by PCs and their phenotypic transformation into myofibroblasts. This condition represents one of the main complications in patients with CKD and affects outcomes and quality of life [86,87]. Despite the importance of this topic, which may overshadow other aspects, studies have also demonstrated the pivotal role of PCs in organ fibrosis [88]. Hypoxic insults represent one of the most important causes of tubulointerstitial injury and peritubular capillary rarefaction, characteristics observed in many fibrotic kidney diseases. Therefore, when hypoxia occurs, PCs begin to produce EPO to fulfill the need for erythropoiesis, but when CKD worsens, these interstitial cells become myofibroblasts and start to deposit ECM to promote tissue repair, losing their ability to produce EPO [89]. During IRI, increased methylation of RAS Protein Activator Like 1 (Rasal1) and Ybx2, the actin alpha 2 (Acta2) repressor gene, is mediated by TGF-β through upregulation of DNA methyltransferase, promoting cell proliferation and α-SMA expression in PCs. Moreover, parallel hypermethylation in the 5′-enhancer and promoter of the EPO gene causes suppression of EPO in myofibroblasts and consequent anemia in CKD. Shih et al. stated that HIF-2α regulates PCs EPO production and showed that in C3H10T1/2 cells, a pericyte cell line, TGF-β1 can suppress HIF-2α expression by activating activin receptor-like kinase-5 (ALK5), resulting in a decreased EPO production [82]. Additionally, Souma et al. showed that reduced EPO production in myofibroblasts may be partially reversed by inactivation of prolyl hydroxylases and consequent activation of HIF pathways [83]. Various studies have demonstrated the importance of HIF genes in the pathogenesis of renal disorders. Some of them stated that HIF activation can reduce the progression rate of kidney diseases and protect cells from ischemic damage, whereas others raised some issues on potential oncogenic effects and deterioration of cardiovascular and kidney functions [90]. Conversely, the effects of HIF stabilization in myofibroblasts or PCs on renal fibrosis are poorly understood [91]. In an animal study, Pan et al. observed the effects of HIFs in PCs on renal pathology with or without fibrotic injury induced by unilateral ureteral obstruction. Gli1^+^ PCs, a small subset of kidney PCs that exhibit mesenchymal stem cell properties, showed increased EPO production, erythropoiesis, and polycythemia but no noticeable change in renal fibrosis after HIF stabilization in VHL or PHD knockout mice. On a cellular level, the mRNA levels of myofibroblast markers, such as collagen type I-alpha1 and type 3-alpha1 chains, (Col1a1, Col3a1), and the levels of Acta2 were not negatively affected by HIF stabilization in PCs/myofibroblasts, underlining its neutral impact on fibrosis [84]. However, further studies are necessary to understand the impact of HIF activation in PCs and in different renal pathological models (i.e., IRI or diabetic nephropathy) because there are numerous other markers of PCs and fibroblasts that manifest as different lineages, such as PDGF-β, CD73, tenascin-C, and smooth muscle myosin protein [92]. Neurologically, the correlation between increased BBB permeability and PCs after stroke led to new discoveries regarding HIF genes. Tsao et al. stated that preventing HIF-1 activation in brain PCs decreases their apoptosis in peri-infarct regions and improves vascular wall coverage. Moreover, the degree of infarction and cerebral edema was much lessened when HIF-1 PC expression was significantly reduced. Hypoxia, in the early phase, has almost no effect on PC survival, but more prolonged damage results in considerable cell death, and the loss of PCs jeopardizes neuronal repair [93]. Additionally, in vivo work showed that PC-targeted loss-of-function mutations in HIF alleviate hypoxia-induced barrier dysfunction but the same mutation had no effect when it occurs in astrocytes [94]. Another study on PC exosome secretion showed that activation of the HIF signaling pathway in PCs stimulated with cobalt chloride promoted wound healing and vessel remodeling. This proangiogenic cell state was inhibited by HIF-1α inhibitors [95]. These data suggest that HIF-1 and PC death are tightly related and entwined with better functional recovery after stroke, although further research is undoubtedly necessary [96]. In myocardial infarction, PCs are among the first cells to experience ischemic insult because of their anatomical location. Lee et al. observed translocation of HIF-1α protein into the PC nucleus after 24 h of 2% hypoxia with increased production of VEGF-A and PDGF and their death approximately four days after exposure to a hypoxic environment [97]. Moreover, sirtuin3 (SIRT3) knockout mice showed PC loss due to impairment of the angiopoietins/Tie 2 and HIF-2α/Notch3 signaling pathways, resulting in a differentiation of PCs in myofibroblasts in the heart [98]. In lung pathology, HIF prolyl hydroxylase domain-2 (PHD2) downregulation and consequent HIF stabilization, leads to an increase in TGF-β production in pulmonary PCs, increasing the risk of developing perivascular fibrosis, vessel dysfunction, and right ventricular hypertrophy. These changes were mediated by higher levels of Ang1 produced by PHD2-deficient ECs, resulting in aberrant Ang/Notch3 signaling. Moreover, a loss of ECs and PCs due to a reduction in hepatocyte growth factor expression was found in HIF-2α knockout mice by Pasupneti et al., which resulted in an emphysematous pathology [99]. Despite the growing interest in the stabilization of HIF signaling pathways through PDH inhibitors, several studies show the complex roles of HIFs in organ pathology, including IRI and fibrosis, and the cell-type dependent functions that HIFs play in the etiology of diseases. In this context, PCs represent one of the main cells in which targeting HIFs would seem useful for developing therapeutic strategies (Figure 4).

## 6. Hints of Potential Therapeutic Strategies

Ischemic preconditioning (IPC), defined as brief and intermittent episodes of controlled ischemic reperfusion before more prolonged ischemia, has been shown to have a protective role against IRI and increased tissue tolerance to hypoxia [94]. IPC increased autophagy pathways in response to IRI [100] and upregulated the expression of miRNA-21 with consequent decreased proinflammatory cytokine production through a HIF-1α dependent mechanism [101]. When compared with wild-type mice subjected to IPC, cardiomyocyte death was reduced in mice expressing the HIF-1α gene, which enhanced heart function, as shown by Cai et al. [102]. In an animal model, the effect of IPC has been evaluated in relation to changes affecting PCs during IRI. The major findings are that IPC could prevent microvascular PC constriction and apoptosis, leading to a decreased infarct size and reduced area of no-reflow, showing that IPC may have a cardioprotective role during myocardial ischemia [103]. According to Wang et al., HIF-1α can promote the transcription of IL-1ra, acting as a protective factor in hepatic IRI through the inhibition of IL-1 signaling pathway, and IPC increases HIF expression by each cycle stimulating IL-1ra release in hepatic tissue [104]. 

HIF-prolyl hydroxylase inhibitors (HIF-PHIs) have become an alternative treatment strategy, compared with erythropoiesis-stimulating agents (ESA), for the treatment of anemia in patients with CKD. They prevent the ubiquitinoylation of HIF-α subunits activating adaptive responses to hypoxia, such as EPO gene transcription. Moreover, some studies support the evidence that HIF stabilization can slow the course of renal disease and protect cells from ischemic injury, but it can also be involved in renal fibrosis [105]. During IRI-related AKI, the use of PHD2 inhibition reduced ROS levels and attenuated oxidative stress. Moreover, the administration of PHD inhibitors hours before IRI could exert a protective role by preventing kidney damage [106,107,108]. PHD inhibitors exert pleiotropic effects. One of them is the regulation of iron metabolism by lowering the levels of hepcidin and ferritin. In chronic inflammatory diseases, such as CKD, the hepcidin and ferritin levels are elevated, leading to an impaired availability of iron, and PHD inhibitors have been found to decrease hepcidin levels in CKD patients [109,110,111]. Moreover, they exert metabolic effects on glucose metabolism, lipid metabolism and cholesterol levels [112,113,114]. HIF stabilization is also essential for the correct function of both innate and adaptive immunity systems. Pharmacological inhibition of PHD2 has increased neutrophilic motility, survival, and inflammation in response to S. Pneumoniae in a model of acute lung injury due to increased glycolytic flux [115], and the inhibition of neutrophils’ PDH3 was associated with decreased inflammation [116]. PHD inhibitors may have different effects on PHD isoforms, which may cause variations in HIF levels and, thus, pleiotropic effects. Further research is needed to elucidate how they can be used in human diseases [117]. Nowadays, there are numerous advancements and potential applications regarding the possibility of modulating HIFs to exert positive effects. For example, in diabetic tissues, insufficient activation of HIF-1α signaling is a fundamental pathogenic factor in the progression of diabetic complications [118], and alterations in PC biology are directly linked to biochemical changes in diabetes [119]. Thus, strategies targeting the modulation of the HIF-1α signaling pathway may be promising as novel treatments in diabetic patients. Moreover, the use of an iron chelator, deferoxamine was shown to correct the impairment of HIF-1α/p300 binding induced by hyperglycemia and normalize the transactivation of HIF-1α [120].

Research has shown that treatment with S-nitrosoglutathione has been found to stabilize HIF-1α and trigger the downstream gene expression of HIF-1α targets, promoting regenerative processes. This leads to functional recovery in animals with mild traumatic brain injury by reducing BBB leakage, reducing expression of MMP-9 and decreasing edema [121,122].

In preclinical studies, tumor development has been seen to be significantly impacted by the inhibition of HIF-1α activity by various methods. Therefore, the use of specific small-molecule inhibitors targeting HIF-1α represents an appealing approach for the development of cancer therapy as shown by Chau et al. [123].

NHE inhibitors have shown a cardioprotective role against IRI due to inhibition of cytosolic Na^+^ accumulation and reduction of Ca^2+^ intracellular overload via reverse mode Na^+^/Ca^2+^ exchange [124,125].

In a renal model of IRI, blocking of PDGFR-β in PCs or VEGFR2 in ECs reduced microvascular rarefaction, inflammation, and interstitial fibrosis through the modulation of PC-EC interactions. Moreover, communication between these types of cells plays a pivotal role during the early stages after a kidney injury. In unilateral ureteral obstruction-induced damage, ECs proliferated to ensure an initial angiogenic response and normal PCs function was important for vascular stabilization, but after four days, PCs began to detach from the vessels and ECs started to become dysfunctional. All these changes are correlated with capillary loss. Lin et al. showed that blocking PDGFR-β and/or VEGFR2 may be a therapeutic strategy for regulating the crosstalk between pivotal cells involved in angiogenesis and response to hypoxic insults [126]. Therefore, the PC–myofibroblast transition might be attenuated by using anti-PDGFR antibodies or imatinib, a tyrosine kinase inhibitor that has been shown to decrease renal fibrosis development after IRI damage [127].

A study indicated that in rat models, the antiplatelet medication cilostazol decreased both the expression and activity of MMP-9, elevated the expression of VEGFR3 and facilitated the detachment of PCs leading to increased angiogenesis and proliferation of PCs, which ultimately helped repair the BBB following ischemic stroke [128].

Moreover, Deguchi et al. showed that the free radical scavenger edaravone has the capability to suppress MMP-9 production, restore the number of PDGFRβ-positive PCs and mitigate the damage at the BBB after ischemic insult in rat models [129].

Atorvastatin, a widely used medication for lipid-lowering, was demonstrated to inhibit Ang2 release and increase the expression of vascular endothelial (VE)-cadherin, resulting in increased vessel maturation and PC coverage [130] (Table 2).

## 7. Conclusions

In this review, we highlighted the central role of pericytes and endothelial cells and their intricate and fine-tuned crosstalk in the regulation of cell responses after ischemia–reperfusion injury. A sensing mechanism can detect variations in O_2_ levels in tissue, leading to HIF stabilization, which is known for its numerous protective properties in preventing organ damage. However, HIF signaling is also involved in the activation of cell death pathways and vascular remodeling and HIF stabilization could therefore have a detrimental impact on the development of fibrotic lesions. In this context, pericytes and their interaction with endothelial cells, during conditions that lead to HIF expression, represent a crucial fascinating topic to discuss further for developing targeted therapies aiming at influencing disease progression and improving treatments of kidney disease and other pathological conditions.

## Figures and Tables

**Table 1:** Collected studies of the paragraph and their major findings.

Organ/Cell Type	Model	Major Findings	References
Kidney	Murine	IRI causes a dysbalance in angiopoietins, loss of ECs, PCs proliferation and fibrosis	Khairoun M et al. [54]
Kidney	Human	IRI causes Increased levels of Ang2 and EC activation	de Vries DK et al. [55]
Kidney	Murine	IRI causes upregulation of the fibrotic marker CD248	Pai CH et al. [57]
Kidney	Murine	Blocking Rho kinase during IRI reduces AKI by inhibiting PCs contraction and increasing medullary blood flow	Freitas F et al. [59]
Kidney	Murine	IRI induces increased expression of PKM2 in PCs, indicating that glycolysis plays a crucial role as a metabolic pathway for PTM	Chen Y et al. [62]
Kidney	Murine	Activation of FAO or inhibition of glycolysis during IRI can prevent AKI to CKD progression	Xu C et al. [63]
Kidney	Swine	PCs are the target of complement activation leading to a profibrotic maladaptive cellular response. C1-INH may be a therapeutic strategy to counteract the development of PMT	Castellano G et al. [64]
Kidney	Murine	PC epigenetic modifications during IRI play a role in their transition towards a profibrotic and proliferative phenotype.	Chou YH et al. [65]
Brain	Murine	PDGFRβ is expressed in PCs in the adult brain indicating that genetic disruption of PDGFRβ signaling leads to a PCs specific injury	Winkler EA et al. [69]
Heart	Murine	After myocardial infarct PCs regulate the induction of genes associated with vascular permeability, extracellular matrix production, basement membrane degradation, and TGF-β signaling	Quijada P et al. [75]
Fibroblasts cell lines	Murine	TGF-β1 can suppress HIF-2α expression by activating ALK5 leading to decreased EPO production	Shih HM et al. [82]
Kidney	Murine	The decreased EPO production in myofibroblasts could be partially reversed by the inactivation of PHD	Souma T et al. [83]
Kidney	Murine	HIF stabilization resulted in increased EPO production and polycythemia but no noticeable change in renal fibrosis.	Pan SY et al. [84]

**Table 2:** Therapeutic interventions and their effects.

Therapeutic Intervention	Effects	References
Ischemic preconditioning	Increase autophagy pathways, decrease proinflammatory cytokine production and prevent microvascular PC constriction and apoptosis	Joo JD et al. [100] Lu N. et al. [101] Jia P. et al. [102] Cai Z. et al. [103]
HIF-prolyl hydroxylase inhibitors	Reduce ROS levels and attenuate oxidative stress and lower the levels of hepcidin and ferritin. Regulate immunity systems and metabolic processes.	Ito M et al. [107] Fleming RE et al. [109] Chen N. et al. [111] Riopel M. et al. [112] Sadiku P. et al. [115] Walmsley SR et al. [116]
Deferoxamine	Correct the impairment of the HIF-1α signaling pathway induced by hyperglycemia	Thangarajah H et al. [120]
S-nitrosoglutathione	Stabilize HIF-1α expression	Khan M. et al. [121] Khan M. et al. [122]
Na^+^/H^+^ exchanger-1 inhibitors	Cardioprotective role	Karmazyn M. [124] Tani M. [125]
PDGFR-β and/or VEGFR2 blocking	Reduce microvascular rarefaction, inflammation, and interstitial fibrosis. Reduce PCs-myofibroblast transition	Lin SL et al. [126] Chen YT et al. [127]
Imatinib	Reduce PC–myofibroblast transition	Chen YT et al. [127]
Cilostazol	Decrease the expression and activity of MMP-9, elevate the expression of VEGFR3 and increase angiogenesis	Omote Y. et al. [128]
Edaravone	Suppress MMP-9 production	Deguchi K. et al. [129]
Atorvastatin	Inhibit Ang2 release and increase the expression of VE-cadherin	Baganha F. et al. [130]
